# Serum Levels of N^ε^-(Carboxymethyl)-Lysine in Chronic Kidney Disease and Type 2 Diabetes Mellitus

**DOI:** 10.3390/biomedicines13071672

**Published:** 2025-07-08

**Authors:** Rositsa Tsekovska, Evan Gatev, Roumyana Mironova, Simona Kerezieva, Siyana Ilieva, Teodora Ilieva, Bilyana Vasileva, Toshimitsu Niwa, Daniela Popova, Vasil Vasilev

**Affiliations:** 1 Roumen Tsanev Institute of Molecular Biology, Bulgarian Academy of Sciences, 1113 Sofia, Bulgaria; evan_gatev@sfu.ca (E.G.); rumym@bio21.bas.bg (R.M.); 2 University Hospital “Tsaritsa Yoanna-ISUL”, Medical University—Sofia, 1431 Sofia, Bulgaria; dr.kerezieva@gmail.com (S.K.); siyana9696@abv.bg (S.I.); t.yo.ilieva@gmail.com (T.I.); b_vasileva_vr@abv.bg (B.V.); danielapopovabg@yahoo.com (D.P.); w_wasilev@yahoo.com (V.V.); 3 Faculty of Medical Sciences, Shubun University, Ichinomiya 491-0938, Aichi, Japan; niwa.t@shubun.ac.jp

**Keywords:** advanced glycation end products, glycoxidation, N^ε^-(carboxymethyl)-lysine, chronic kidney disease, diabetes mellitus

## Abstract

**Background**: N^ε^-(carboxymethyl)-lysine (CML) is formed in the human body by non-enzymatically driven reactions including glycation, oxidation, and lipoxidation. CML is a ubiquitous product of normal physiology, but its levels are increased under disease conditions like chronic kidney disease (CKD) and diabetes mellitus (DM). Free CML is eliminated from the human body mainly through kidney excretion, and its accumulation in the kidney tissue is linked to CKD pathogenesis. **Aim**: The main goal of this study was to evaluate the relative contribution of CKD and Type 2 DM (T2DM) to the accumulation of CML in patients’ sera. **Methods**: The study included 22 patients with CKD without DM, 55 with CKD and comorbid T2DM, and 21 with T2DM without CKD. Serum CML levels were measured by ELISA. The Kruskal-Wallis test was used to detect differences among groups. Spearman correlation analysis was performed, and the one-tailed Dunn test was considered to indicate statistical significance at *p* < 0.05. **Results**: The median serum CML levels (CKD, 658.4 ± 434.3 ng/mL; CKD + T2DM, 431.3 ± 327.9 ng/mL; T2DM, 273.9 ± 134.2 ng/mL) differed significantly (*p* < 0.05) among the three patient groups. A positive correlation was observed between serum CML and microalbuminuria (*p* = 0.004; *r* = 0.58), proteinuria (*p* = 0.002; *r* = 0.6), and age (*p* = 0.007; *r* = 0.52) only in the CKD patients. In all T2DM patients, independent of CKD status, serum CML correlated negatively (*p* < 0.05) with postprandial glucose and duration of diabetes, while its correlation with fasting glucose and HbA1c was negative only in the T2DM cohort without CKD. **Conclusions**: In patients with CKD, higher levels of CML were observed compared to those with T2DM. Serum CML correlated positively with proteinuria, albuminuria, and patient age in non-diabetic CKD patients, and negatively with blood glucose, HbA1c, and DM duration of T2DM in patients without CKD.

## 1. Introduction

Chronic kidney disease (CKD) is a progressive condition, characterized by a gradual decline in kidney function, which can lead to end-stage renal disease (ESRD), requiring dialysis or transplantation. It affects over 800 million individuals globally, representing more than 10% of the human population [1]. CKD is characterized by a progressive decrease in the glomerular filtration rate (GFR), i.e., to less than 60 mL/min per 1.73 m^2^, or by the presence of proteinuria [2]. The prevalence of CKD is notably higher among individuals with comorbid conditions such as diabetes and hypertension. Studies on CKD are considered a topical issue due to its impact on patients’ quality of life and its burden on healthcare systems, with costs escalating with disease progression, comorbidities, and complex care needs [3,4,5]. Early diagnosis and intervention are crucial to prevent or slow the progression of CKD.

The pathophysiology of CKD involves a variety of mechanisms, including metabolic and cellular dysfunction, hemodynamic changes, inflammatory processes, and structural alterations within the kidneys. The final histological changes, including glomerulosclerosis, tubular atrophy, arteriolohyalinosis, and interstitial fibrosis reflect the underlying causes of CKD (e.g., diabetes, hypertension, glomerulonephritis), driving the progression of kidney dysfunction. The primary cell types affected in glomerulosclerosis include podocytes, endothelial cells, and mesangial cells. Injury to podocytes, which form the filtration barrier, results in the effacement and loss of podocyte foot processes. Damage to endothelial cells disrupts the normal glomerular hemodynamics while mesangial cells proliferate in response to injury, thus contributing to matrix expansion and scarring. This process is often driven by inflammatory cytokines such as transforming growth factor beta (TGF-β), which promotes further podocyte and endothelial dysfunction. Tubular atrophy primarily involves tubular epithelial cells. These cells experience apoptosis and senescence due to chronic injury by factors like oxidative stress and inflammation. The loss of functional epithelial cells results in reduced tubular mass and function. Additionally, myofibroblasts, derived from activated fibroblasts or through epithelial-to-mesenchymal transition (EMT) of tubular cells, contribute to the fibrotic process by producing excessive extracellular matrix (ECM). Inflammatory cells, particularly macrophages, also play a role in promoting tubular cell death through cytokine release. Interstitial fibrosis is characterized by the activation of fibroblasts and their differentiation into myofibroblasts, excessive ECM production, and scarring within the renal interstitium. Inflammatory cells, including macrophages and T lymphocytes, infiltrate the interstitium and secrete pro-fibrotic mediators that promote fibrosis. In addition, tubular epithelial cells can undergo EMT, thus expanding the pool of myofibroblasts and promoting further fibrotic changes [6,7,8,9,10,11,12]. The interplay between inflammation, cytokine signaling, and oxidative stress creates a detrimental environment priming renal cell injury in CKD patients and leading to a progressive loss of kidney function.

Diabetes mellitus (DM) is a metabolic disease, clinically characterized by hyperglycemia. There are two main types of diabetes, type 1 (T1DM) and type 2 (T2DM), resulting from defective insulin secretion and/or activity. The pathogenesis of the two types of diabetes is different, and each has a specific etiology, manifestation, and treatment. Diabetes is a global epidemic. According to the International Diabetes Federation, in 2021, the number of affected adults worldwide was 537 million. Diabetes is associated with macrovascular complications such as coronary heart disease, ischemic disease, and peripheral artery disease, whereas microvascular diabetic complications comprise nephropathy, retinopathy, and neuropathy [13]. These vascular alterations are associated with structural and functional changes in tissues and organs that can lead to multi-organ dysfunction, sometimes with lethal outcome. In diabetes, hyperglycemia is the primary driver for the production of advanced glycation end products (AGEs) through glycation, creating a vicious cycle of metabolic damage. AGE accumulation under hyperglycemic conditions promotes excessive oxidative stress, activates inflammatory pathways, and accelerates pathological changes, contributing to diabetic complications, including diabetic nephropathy (DN) [14,15,16,17]. In this complex cascade, AGEs serve as critical products driving DN toward CKD.

AGEs are harmful compounds formed through non-enzymatic reactions between carbonyl compounds (among which are reducing sugars like glucose) and primary amines including amino acids, proteins, aminolipids, etc. The detrimental effect of AGEs on kidney tissue is caused by modifications and crosslinking of proteins or by binding to the cellular receptor for AGEs (RAGE) [18,19]. AGEs contribute to oxidative stress by generating reactive oxygen species (ROS) upon interaction with RAGE. This interaction activates various intracellular signaling pathways that lead to increased ROS production. In turn, elevated oxidative stress can damage cellular structures, thus exacerbating tissue injury and promoting the progression of chronic diseases, including CKD [20,21,22]. AGEs also play a significant role in inflammatory processes [23]. They stimulate the expression of pro-inflammatory cytokines such as tumor necrosis factor alpha (TNF-α) and interleukin 6 (IL-6) through RAGE-mediated signaling pathways. The chronic presence of AGEs may provoke a sustained inflammatory response and overexpression of mineralcorticoid receptors, contributing to the development of fibrosis and to decline in renal function in CKD patients [24,25]. The interaction of AGEs with RAGE up-regulates nuclear factor kappa B (NF-κB), a key regulator of inflammation [26], and activates signaling cascades that enhance cytokine production [27]. The resulting increase in the local concentration of pro-inflammatory cytokines not only promotes inflammation but also affects systemic responses, linking AGEs to various age-related diseases such as cardiovascular disorders and diabetes.

N^ε^-carboxymethyl-lysine (CML) is a major product of advanced glycation that plays a crucial role in oxidative stress and long-term protein damage under different conditions like aging, atherosclerosis, and diabetes [28,29,30]. CML is formed via multiple biochemical pathways. These pathways include the oxidative degradation of Amadori products, reactions with reactive dicarbonyl compounds like glyoxal and methylglyoxal, lipid peroxidation, and serine oxidation [31,32,33]. CML is of particular clinical relevance for assessing the progression of the renal impairment in diabetic patients, because serum CML levels increase in DM patients with CKD in parallel with reduced GFR, increased oxidative stress, and the severity of the nephropathy [34,35,36,37]. Increased CML levels are also associated with an increase in NF-κB activity, and respectively with stimulation of inflammatory pathways, increased cytokine production, and further exacerbation of kidney tissue damage [38,39]. CML promotes lipid accumulation in kidney cells by up-regulating the expression of genes involved in the synthesis and absorption of cholesterol, which is a critical contributor to structural changes and kidney dysfunction [40]. Elevated CML levels are linked to enhanced production of TGF-β, a key cytokine involved in fibrogenesis. Activation of the TGF-β signaling pathway enhances the synthesis of extracellular matrix components that are essential for the development of renal fibrosis [41,42].

CML is a ubiquitous product of normal human physiology, as well as of pathological states like hyperglycemia, oxidative stress, and chronic inflammation. Therefore, CML appears an attractive target for research and clinical evaluation, especially under conditions of glycoxidative stress, which is enhanced in patients with CKD and DM. We hypothesized that comorbidity of CKD with T2DM would influence serum CML levels in such patients, in either a cumulative or a suppressive manner. To test this hypothesis, the current study aimed to evaluate the relative contribution of CKD and T2DM to the accumulation of CML in patients’ sera, as well as to reveal correlations of serum CML levels with markers of kidney dysfunction and diabetes.

## 2. Materials and Methods

### 2.1. Study Design

A single-center, cross-sectional study was performed to assess the serum CML levels in patients recruited from the Clinic of Nephrology and from the Clinic of Endocrinology and Metabolic Diseases for Treatment of Metabolic Disorders of the University Hospital “Tsaritsa Yoanna-ISUL” in Sofia (Bulgaria) during the period of March 2023 to February 2024.

### 2.2. Patients and Sample Collection

The study included 22 patients with CKD without DM, 55 patients with CKD and comorbid T2DM, and 21 patients with T2DM only. All participants underwent routine clinical examinations, anthropometric measurements, and laboratory tests upon admission. Estimated GFR (eGFR) was calculated using the Modification of Diet in Renal Disease (MDRD) formula based on the serum creatinine concentration. CKD staging was performed according to the recommendations of The Kidney Diseases Improving Global Outcomes (KDIGO) group, 2012 [2].

Blood and urine samples were collected from patients as part of routine clinical procedures in the course of ongoing nephroprotective treatment (RAS-inhibitors or/and SGLT2-inhibitors) in all cases where no contraindications were present. The patient cohorts consisted of individuals admitted for various reasons: for initial diagnosis of the disease, for management of disease-related complications, and for routine follow-up appointments, related to patients’ pre-existing conditions. This approach ensured a diverse representation of patients at different stages of the disease progression. The following inclusion criteria were applied: age between 18 and 75; and clinical and laboratory evidence of T2DM and various stages of CKD, according to eGFR, ranging from G1 to G5. Among the CKD patients with and without T2DM, there were no kidney transplant recipients. Exclusion criteria were: cardiovascular accident in the last 3 years; New York Heart Association (NYHA) class III and IV heart failure; cerebrovascular accident in the last 3 years; infectious and neoplastic diseases; amyloidosis; pregnancy; or corticosteroid therapy.

### 2.3. Measurement of Serum CML

CML was quantified by competitive Enzyme-Linked Immunosorbent Assay (ELISA). The coating antigen, bovine serum albumin (BSA) (Sigma-Aldrich, St. Louis, MO, USA, A7960) modified with AGEs (AGEs-BSA), was prepared by incubating 10 mg/mL BSA with 0.1 M glucose in the presence of 0.02% sodium aside over 3 months, at 37 °C, in a laboratory thermostat incubator (Wincom Company Ltd., Changsha, China). ELISA plates (Corning Inc., Corning, NY, USA, 2797) were coated with 100 μL/well AGEs-BSA at a concentration of 1 μg/mL, in 50 mM carbonate buffer, pH 9.5, and incubated overnight at 37 °C. On the next day, the coating solution was discarded and the plate was washed two times with 200 μL/well 0.05% Tween in PBS, pH 7.4, and once with 200 μL/well PBS, pH 7.4. CML (Iris Biotech GmbH, Marktredwitz, Germany, HAA2950) standard solutions were prepared in PBS, pH 7.4, in the concentration range from 1 ng/mL to 200 ng/mL. Then, 50 μL serial dilutions of the CML standard and patients’ sera were added to the plate wells and mixed with 50 μL 0.25% BSA, supplemented with an anti-CML and anti-mouse IgG antibodies, at titers 1:1000 and 1:5000, respectively. The anti-CML [CML126] ab125145 and the rabbit anti-mouse IgG H&L (HRP) ab6728 antibodies were from Abcam Ltd. (Cambridge, UK). Plates were incubated with the two antibodies for 2 h at 37 °C and washed as described above, and the color reaction was developed with 100 μL/well 100 mM citrate buffer, pH 6.0, containing 1 mg/mL *o*-phenylenediamine (Sigma-Aldrich, St. Louis, MO, USA, P9029). After 15 min incubation at room temperature, the developing reaction was stopped by the addition of 100 μL/well 0.8 M H_2_SO_4_, and absorption at 490 nm was measured with a plate reader Bio-TEK ELx800 (BioTek Instruments, Inc., Winooski, VT, USA). A 4-Parameter Logistic curve (4PL) was applied for data processing.

### 2.4. Statistical Analysis

Data were tested for normality of the distribution with the Anderson-Darling test. All data were not normally distributed, so ANOVA analysis was not appropriate. Rather, we used the Kruskal-Wallis test to detect differences among groups, followed by pairwise group comparisons with the Dunn test. One-tailed Dunn test was considered to indicate statistical significance at *p* < 0.05. We performed a power analysis on patient data with the software G*Power 3.1 [43] under the assumption that the patient data were log-normally distributed. The variables were summarized as medians ± MAD (median absolute deviation). In order to explore the CML biomarker potential, we conducted Spearman correlation analysis of serum CML levels with patients’ clinical and biochemical characteristics. The open source R-software (R Found. Stat. Comp., Vienna, Austria), version 4.4.2, was used for the statistical analysis.

## 3. Results

### 3.1. Characteristics of the Three Groups of Patients

#### 3.1.1. Age

The median patient age in the three groups did not differ significantly: of the patients with CKD without DM, 52.5 ± 6.5 years (range: 34–74 years); of the T2DM patients with CKD, 65 ± 7 years (range: 41–75 years); and of the patients with T2DM without CKD, 52 ± 6 years (range: 33–73 years).

#### 3.1.2. Sex

Among the group of patients with CKD without DM, 54.55% (12) were male. In the group of T2DM patients with CKD, 50.9% (28) were male, and in the group of T2DM patients without CKD, 52.4% (11) were male.

#### 3.1.3. Fasting Glucose

The patients within each group were distributed, based on fasting glucose (FG) levels, to match the World Health Organization (WHO) and the International Diabetes Federation (IDF) criteria. The FG values varied across the three groups according to their distinct clinical profiles. The group of patients with CKD without DM demonstrated FG values predominantly within the normal range, which indicated the absence of diabetes-related glucose dysregulation. In contrast, the patients with CKD and T2DM exhibited elevated FG levels, characteristic of impaired glucose metabolism. The group of T2DM patients without CKD also showed elevated FG levels, i.e., comparable to or slightly lower than those observed in the T2DM patients with CKD, as a result of diabetes management in the absence of kidney impairment. The patients’ distribution according to the FG levels is summarized in Table 1.

### 3.2. Characteristics of the CDK Patients with and Without DM

#### 3.2.1. eGFR

CKD severity in CDK patients with and without DM was classified according to the KDIGO standard, based on the eGFR and the presence of albuminuria.

The classification of the CKD patients without DM according to eGFR was as follows: 3 patients (13.6%) with eGFR ≥ 90 mL/min (G1); 8 patients (36.4%) with eGFR 60–89 mL/min (G2); 5 patients (22.7%) with eGFR 45–59 mL/min (G3a); 2 patients (9.1%) with eGFR 30–44 mL/min (G3b); 2 patients (9.1%) with eGFR 15–29 mL/min (G4) and 2 patients (9.1%) with eGFR < 15 mL/min (G5).

**Figure 1:**
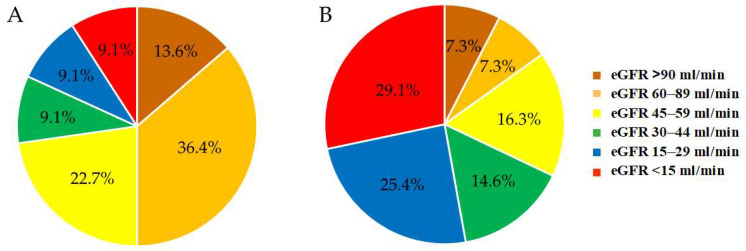
Distribution of the patients with CKD without DM (**A**) and of the CKD patients with T2DM (**B**) according to eGFR.

Depending on the eGFR value, the patients with CKD and T2DM were divided into the following groups: 4 patients (7.3%) in the group with eGFR ≥ 90 mL/min (G1); 4 patients (7.3%) in the group with eGFR 60–89 mL/min (G2); 9 patients (16.3%) in the group with eGFR 45–59 mL/min (G3a); 8 patients (14.6%) in the group with eGFR 30–44 mL/min (G3b); 14 patients (25.4%) in the group with eGFR 15–29 mL/min (G4) and 16 patients (29.1%) in the group with eGFR < 15 mL/min (G5). The distribution of the two groups of CKD patients, with and without DM, according to eGFR is presented in Figure 1. The eGFR profiles of the two groups of CKD patients differed toward a significantly deteriorated renal function in the diabetics compared to the non-diabetics.

#### 3.2.2. Albuminuria

Depending on the degree of albuminuria (mg/24 h), the CKD patients without DM were divided into the following groups: 4 patients (18.2%) with normal or mildly elevated albuminuria (normoalbuminuria) (<30 mg/24 h) (A1); 7 patients (31.8%) with moderately elevated albuminuria (microalbuminuria) (30–300 mg/24 h) (A2); 11 patients (50.0%) with significantly elevated albuminuria (macroblbuminuria) (>300 mg/24 h) (A3).

**Figure 2:**
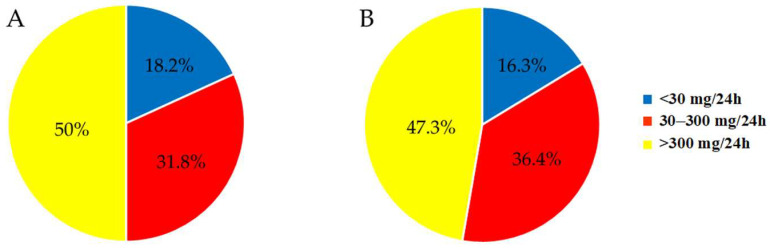
Distribution of the CKD patients without DM (**A**) and of the patients with CKD and T2DM (**B**) according to the stage of albuminuria.

The distribution of the CKD patients with T2DM according to albuminuria (mg/24 h) was as follows: 9 patients (16.3%) with normoalbuminuria (<30 mg/24 h) (A1); 20 patients (36.4%) with microalbuminuria (30–300 mg/24 h) (A2); 26 patients (47.3%) with macroblbuminuria (>300 mg/24 h) (A3). The results are presented in Figure 2.

#### 3.2.3. Proteinuria

Depending on the proteinuria levels, based on 24-h urine collection, patients with CKD without DM were divided into the following groups: 4 patients (18.2%) with normal, physiological proteinuria (<0.15 g/24 h), 5 patients (22.7%) with mild proteinuria (0.15–0.5 g/24 h), 10 patients (45.5%) with moderate proteinuria (0.5–3.5 g/24 h) and 3 patients (13.6%) with severe or nephrotic-range proteinuria (>3.5 g/24 h).

**Figure 3:**
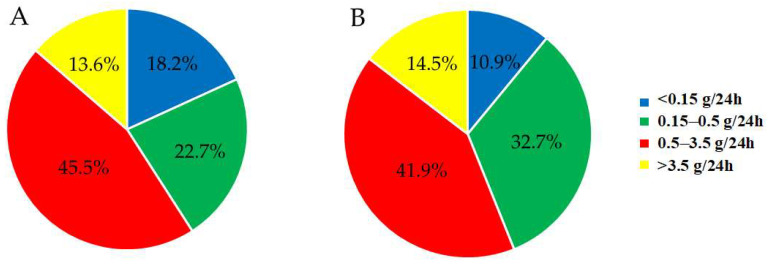
Distribution of the patients with CKD without DM (**A**) and of the CKD patients with T2DM (**B**) according to the proteinuria levels.

The distribution of the CKD patients with T2DM was as follows: 6 patients (10.9%) with normal proteinuria (<0.15 g/24 h), 18 patients (32.7%) with mild proteinuria (0.15–0.5 g/24 h), 23 patients (41.9%) with moderate proteinuria (0.5–3.5 g/24 h) and 8 patients (14.5%) with severe proteinuria (>3.5 g/24 h). The distribution of the two groups of CKD patients, with and without DM, according to proteinuria, is presented in Figure 3.

The two CKD patient groups demonstrated similar distributions, based on both albuminuria (Figure 2) and proteinuria (Figure 3), despite the more than twice smaller sample size of the CKD cohort without DM. These data indicate that CKD, whether caused by diabetes or other conditions, leads to a comparable risk of protein leakage, reinforcing the hypothesis that CKD itself is a major determinant of albuminuria and proteinuria.

### 3.3. Characteristics of the T2DM Patients with and Without CKD

#### 3.3.1. Duration of Diabetes

The duration of diabetes differed between the T2DM patients with and without CKD, reflecting a trend toward longer disease duration in those with kidney impairment. The T2DM patients with CKD had a mean DM duration of 156 ± 72 months (range 12 to 600), while those with T2DM without CKD had a significantly shorter mean DM duration of 60 ± 48 months (range 12 to 444). Despite the large variation in DM duration within each group, the difference between the two groups reached statistical significance (*p* = 0.014). These data reveal the duration of diabetes as one of the contributing factors to the development of CKD, together with other clinical, metabolic, and lifestyle impacts.

#### 3.3.2. Postprandial Glucose

The distribution of the T2DM patients with and without CKD, based on the postprandial glucose (PPG) levels measured 2 h after meal, was prepared according to the IDF criteria. CKD alone had a negligible impact on the PGG levels in the studied cohorts. The distribution of the PPG levels between the T2DM patients with CKD and those without CKD was similar, as visible from Table 2, pointing to hyperglycemia as the key determinant of blood glucose levels after meal.

### 3.4. Power Analysis

To evaluate the statistical power to detect differences between groups, we plotted the power against the size of the effect for our patient samples sizes at *p*-value of 0.05 (Supplementary Figure S1). As expected, given the modest sample sizes of our pilot study, we were under-powered to detect small sized effects. However, the power was adequate to detect the larger effects that were present in our data. For example, calibrating the log-normal distribution to the patient data, the normalized difference in the serum CML between the groups CKD and T2DM was 0.89, corresponding to a statistical power of 0.87.

### 3.5. Serum CML Levels in the Three Groups of Patients

CML is one of the major products formed in the human body as result of non-enzymatically driven reactions such as oxidation, glycation, and lipoxidation [31,32,33]. Therefore, the measurement of the serum CML levels in this study provides insights into the complex effect of hyperglycemia and renal dysfunction on the glycoxidative and lipoxidative stress in humans under the respective clinical conditions.

**Figure 4:**
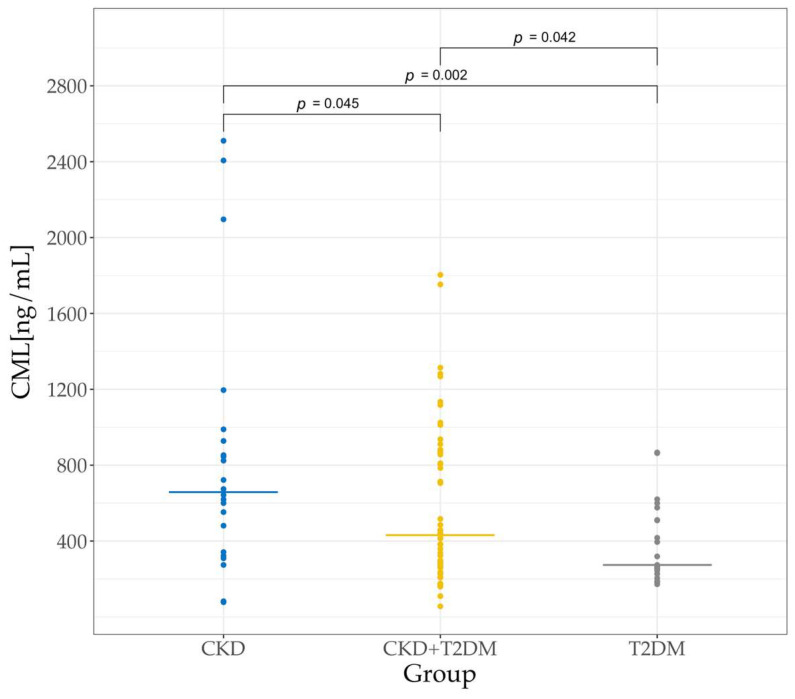
Median serum CML levels in the three groups of patients.

We observed a statistically significant difference in the CML levels among the three groups of patients (Kruskal-Wallis, *p* = 0.017). In the patients with CKD without DM, the serum CML levels reflected the impact of the renal dysfunction on CML accumulation in the absence of hyperglycemia-driven glycoxidation processes. The median serum CML level in these patients (658.4 ± 434.3 ng/mL) was significantly higher (*p* < 0.05) than in the other two patient groups (Figure 4).

In patients with CKD and T2DM, the median serum CML level of 431.3 ± 327.9 ng/mL was significantly lower than in the CKD patients without DM (*p* = 0.045) but significantly higher than in the T2DM patients without CKD (*p* = 0.042). The differences remained statistically significant at 5% after a Benjamini-Hochberg multiple-test correction, except for the difference between the patients with CKD and T2DM versus the T2DM patients without CKD (adjusted *p*-value of 0.06). These data show that patients with CKD had significantly higher levels of serum CML than CKD patients with comorbid diabetes.

In patients with T2DM without CKD, serum CML levels could be used to assess the contribution of hyperglycemia to CML formation in the context of a preserved renal function. In these patients, we measured the lowest median CML concentration (273.9 ± 134.2 ng/mL) among the three patient groups.

### 3.6. Correlation of Serum CML Levels with Patients’ Age and Markers of Kidney Disease

**Figure 5:**
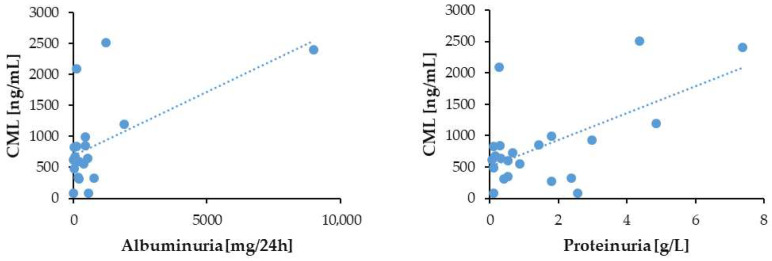
Correlation of the serum CML with albuminuria and proteinuria in the CKD patients without DM.

Microalbuminuria is an early indicator of kidney damage, often detected before a significant proteinuria occurs. In patients with CKD without DM, we observed a significant positive correlation between serum CML levels and both albuminuria (*p* = 0.004; *r* = 0.58) and proteinuria (*p* = 0.002; *r* = 0.6) (Figure 5). No correlation of CML with these markers of renal impairment was present in the group of CKD patients with T2DM (albuminuria, *p* = 0.396; *r* = −0.04; proteinuria, *p* = 0.141; *r* = −0.16). These data indicate that elevated CML levels in blood tend to mirror the progression of the kidney damage in CKD patients, a trend which is perturbed when CKD develops under diabetic conditions.

**Figure 6:**
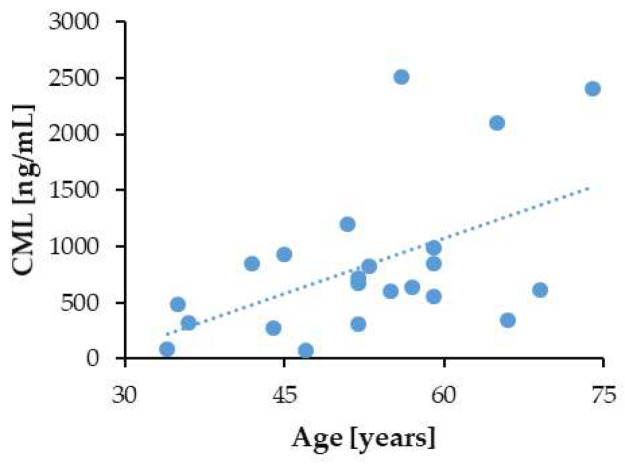
Correlation of the serum CML with age in the CKD patients without DM.

AGEs, including CML, accumulate in the human body with advancing age [44,45,46]. In compliance with this fact, in the group of the non-diabetic patients with CKD, we found a positive correlation between serum CML levels and patient age (*p* = 0.007; *r* = 0.52) (Figure 6). Again, the diabetic state violated the correlation of the CML levels with patient age in the group of the CKD patients with T2DM (*p* = 0.181; *r* = −0.13). There was also no significant correlation between serum CML and age in T2DM patients without CKD (*p* = 0.338; *r* = −0.1).

Although the eGFR profiles of both CKD patient groups were quite different (Figure 1), we did not observe any significant correlation of the serum CML levels with eGFR, either in the CKD without DM (*p* = 0.323, *r* = 0.2) or in the CKD with T2DM (*p* = 0.372, *r* = 0.05) patient groups.

### 3.7. Correlation of Serum CML Levels with Duration of Diabetes and Glycemic Markers

**Figure 7:**
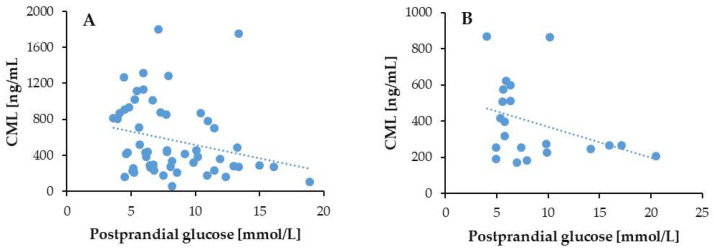
Correlation of serum CML with PPG in T2DM patients with (**A**) and without (**B**) CKD.

We observed a negative correlation between the serum CML levels and postprandial glucose, regardless of CKD status: in the T2DM patients with CKD (*p* = 0.01; *r* = −0.31) and in the T2DM patients without CKD (*p* = 0.05; *r* = −0.36) (Figure 7).

**Figure 8:**
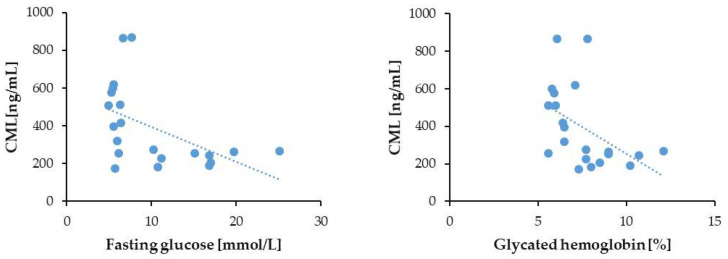
Correlation of serum CML with FG and HbA1c in the T2DM patients without CKD.

The correlation of the serum CML levels with fasting glucose (*p* = 0.008; *r* = −0.52) and glycated hemoglobin (HbA1c) (*p* = 0.005; *r* = −0.55) in the T2DM group without CKD was also negative (Figure 8), whereas in the group of T2DM patients with CKD, there was a weak and non-significant (*p* = 0.172; *r* = −0.13) trend of fasting glucose to correlate negatively with the serum CML levels. No clinical data were available for HbA1c levels in the T2DM patients with CKD.

**Figure 9:**
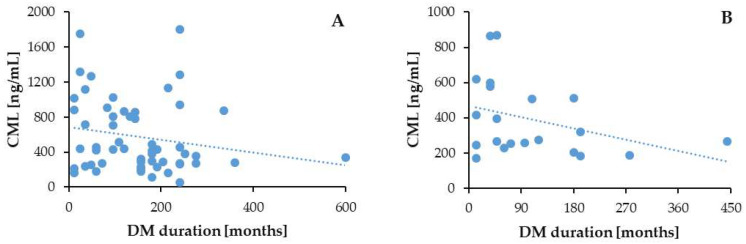
Correlation of CML with DM duration in T2DM patients with (**A**) and without CKD (**B**).

In the T2DM patients without CKD, the serum CML levels tended to correlate negatively also with the duration of diabetes (*p* = 0.05; *r* = −0.36), whereas in the patients with both disorders, i.e., T2DM and CKD, the negative correlation trend was weaker and less significant (*p* = 0.08; *r* = −0.19) (Figure 9).

## 4. Discussion

Serum CML levels are the net result of CML accumulation in the blood plasma and its elimination from the circulation through different pathways. CML can be ingested with food in both free and protein-bound forms, with free CML being more readily absorbed in the gastrointestinal tract [47,48]. Another source of plasma CML is the intracellular degradation of CML-modified proteins by the proteasome [49], the ubiquitin-proteasome system [47,50], and the autophagy pathway [51], followed by a release of the resulting free and peptide-bound CML in the circulation. Some contribution to plasma CML levels also may be made through the glycation of serum proteins (mainly albumin) and of circulating free lysine [52]. Marked increases of glycation adducts including CML in physiological fluids are observed in diabetes and uremia, associated with vascular complications in affected patients [53].

The kidneys play a pivotal role in the excretion of free CML, with animal studies showing that up to 29% of the dietary CML can be recovered in urine [54]. However, the efficiency of the urinary excretion can be influenced by the complexity of the dietary CML compounds [47,54] and renal functionality. Residual renal function (RRF) has been shown to be inversely related to CML levels in the body. Patients with reduced RRF show higher CML concentrations, indicating that efficient kidney function is essential for CML clearance [55]. Corroborating this, the CKD patients in our study, comprising non-diabetics and diabetics, demonstrated significantly higher serum CML concentrations compared to the diabetic patients with normal renal function. Moreover, in the non-diabetic CKD patients, CML correlated positively with the proteinuria (*p* = 0.002; *r* = 0.6) and albuminuria (*p* = 0.004; *r* = 0.58) markers of kidney dysfunction. Here, it is worth noting that in our study, the distribution of the CKD patients with and without diabetes according to albuminuria (Figure 3) and proteinuria (Figure 4) was very similar. Studies by others have also reported elevated serum CML levels in CKD patients and diabetics [56,57,58] and on an association of plasma CML with proteinuria and glomerular abnormalities [37,59]. We measured the highest level of CML in the sera of the non-diabetics with CKD and the lowest in the sera of the diabetics without CKD, which points to kidney injury, and not diabetes, as the primary driver of CML accumulation in the blood.

Although oxidative stress and inflammation in diabetes are expected to increase the AGEs (including CML) burden [14,28], the two T2DM patient groups in our study demonstrated a significant decrease of serum CML concentrations compared to the non-diabetic CKD patients. This result is not surprising, as enhanced oxidative and inflammatory reactions under diabetic conditions may trigger additional pathways of CML clearance, including innate and adaptive immune responses. For instance, the binding of CML to RAGE on immune cells may trigger inflammatory responses [60]. Moreover, the immune system can generate specific antibodies (Abs) against CML [61]. Reddy et al. (1955) showed that CML is one of the major epitopes of AGEs-modified proteins [62], and a subsequent study demonstrated that the level of autoantibodies against CML may vary depending on the disease context and treatment [63]. Vay et al. (2000) observed 289 diabetic patients and 120 healthy individuals for serum Abs against CML and found an increased titer of anti-CML Abs of IgG-isotype in the sera of diabetic patients compared to controls (*p* < 0.0001) [64]. In a recent study including 21 non-diabetic CKD and 23 diabetic CKD patients, we also observed a 25% increase (*p* = 0.04) in the titer of the anti-AGEs Abs in the sera of the diabetics compared to the non-diabetics [65]. Based on these experimental data, we propose that enhanced neutralization of circulating CML antigens by anti-CML antibodies in diabetics may contribute to lower serum CML levels in such patients as compared to non-diabetic CKD patients.

Multiple studies have demonstrated that CML levels increase in older subjects [44,45,46]. We also observed a positive correlation (*p* = 0.007; *r* = 0.52) between serum CML and age among the non-diabetic CKD patients. The lack of such correlation in the two diabetics groups, i.e., T2DM with CKD (*p* = 0.181; *r* = −0.13) and T2DM without CKD (*p* = 0.338; *r* = −0.1), possibly resulted from the above proposed, more robust involvement of immune responses in CML clearance under diabetic conditions. It should be considered that in contrast to free CML in blood, CML bound to tissue proteins becomes immunogenic, and that in non-diabetics with CKD, CML accumulates mainly in the renal tissue, while in diabetics, many other tissues can be affected as a result of enhanced glycoxidative stress [66,67]. Thus, the body’s overload with CML antigens in diabetics may provoke enhanced immunoreactivity to CML with advancing age, as compared to non-diabetics with CKD only. Note that although not statistically significant, the correlation of serum CML with age in the two T2DM groups demonstrated a negative trend. Moreover, we observed a negative correlation trend of serum CML levels with the duration of diabetes in both T2DM patient groups, i.e., T2DM without CKD (*p* = 0.05; *r* = −0.36) and T2DM with CKD (*p* = 0.08; *r* = −0.19). Long-standing diabetes is not necessarily associated with advanced age of patients, but in our study, there was a positive correlation between patient age and DM duration in both T2DM cohorts, i.e., T2DM with CKD (*p* = 0.01; r = 0.3) and T2DM without CKD (*p* = 0.04; r = 0.38). Shibayama et al. (1999) reported that in streptozotocin-induced diabetic rats, the autoantibody activity against CML increased with prolongation of the diabetic state [63]. In summary, it would appear that persistent diabetes results in decreased serum CML levels, most probably due to the neutralization of circulating CML by the immune system.

We also investigated the correlation of serum CML levels with glycemic markers, i.e., fasting glucose, postprandial glucose, and HbA1c. In both T2DM patient groups, the serum CML levels correlated negatively with the postprandial glucose measured 2 h after a meal: T2DM with CKD (*p* = 0.01; *r* = −0.31) and T2DM without CKD (*p* = 0.05; *r* = −0.36). Fasting blood glucose also showed a negative correlation with CML in the diabetics without CKD (*p* = 0.008; *r* = −0.52), whereas in diabetics with CKD, the negative correlation trend was weaker and insignificant (*p* = 0.08; *r* = −0.19). In our study, clinical data for HbA1c were available only for the T2DM patients without CKD. These data also demonstrated a negative correlation of HbA1c with serum CML levels (*p* = 0.005; *r* = −0.55). Many studies have shown a positive correlation of CML levels with diabetic complications, including retinopathy [68], acute coronary syndrome [69] and nephropathy [70]. However, in early-stage diabetic nephropathy, CML was not significantly correlated with the urinary albumin to creatinine ratio, indicating that CML might not be a reliable marker for early nephropathy [71]. Studies also have shown that CML levels do not significantly correlate with fasting blood glucose or HbA1c levels in diabetic patients. For instance, in one study, fasting blood glucose (*p* = 0.12) and HbA1c (*p* = 0.65) did not significantly affect the CML levels in patients with diabetic retinopathy [68]. Another study found no significant association between CML levels and HbA1c among T2DM patients with ischemic heart disease [72]. These findings, together with our observations, indicate that while CML is a useful marker for certain diabetic complications, it may not be a reliable marker for glycemic control.

This study examined the hypothesis that comorbidity of CKD with T2DM would influence serum CML levels in either a cumulative or suppressive manner. As seen from the results, both effects were observed. Relative to T2DM, CKD comorbidity had a cumulative impact on serum CML levels. In contrast, relative to CKD, T2DM comorbidity suppressed the serum CML levels. In future studies, we intend to test the proposed role of adaptive immunity through these effects by evaluating titers of anti-CML antibodies in patient sera. In this context, it is of particular relevance to conduct a similar study with T1DM patients. We hypothesize that in such patients, the activation of the immune system due to the autoimmune character of this disorder might further suppress serum CML levels relative to T2DM. To avoid the limitation of the small patient groups in the current study, we are now designing a similar prospective multicenter study which will include additional patients with T1DM.

## 5. Conclusions

In CKD patients with and without T2DM, higher levels of CML were measured as compared to patients with T2DM only. Also, CML levels in sera could be used to distinguish comorbidity of CKD with T2DM from the CKD and T2DM states alone. Serum CML correlated positively with proteinuria, albuminuria, and patient age of non-diabetic CKD patients, and negatively with blood glucose, HbA1c, and DM duration of T2DM patients without CKD.

## Figures and Tables

**Table 1:** Distribution of patients based on fasting glucose.

Study Group	FG < 6.1 mmol/L	FG 6.1–6.9 mmol/L	FG ≥ 7.0 mmol/L
CKD^+^ DM^−^	86.36% (19)	9.09% (2)	4.55% (1)
CKD^+^ T2DM^+^	25.45% (14)	14.55% (8)	60% (33)
CKD^−^ T2DM^+^	33.33% (7)	19.05% (4)	47.62% (10)

**Table 2:** Distribution of the T2DM patients with and without CKD based on PPG levels.

Study Group	PPG < 7.8 mmol/L	PPG 7.8–11.1 mmol/L	PPG ≥ 11.1 mmol/L
T2DM^+^ CKD^+^	56.36% (31)	23.64% (13)	20% (11)
T2DM^+^ CKD^−^	61.9% (13)	19.05% (4)	19.05% (4)

To readers’ convenience, all patients’ data are summarized in Supplementary Table S1.

## Data Availability

All research data are available upon request at E-mail: rcekovska@gmail.com.

## Supplementary Materials

The following supporting information can be downloaded at: https://www.mdpi.com/article/10.3390/biomedicines13071672/s1, Figure S1: Statistical power of Wilcoxon-Mann-Whitney *U*-test for different effect sizes, given samples sizes N1 = 22 (CKD) and N2 = 21 (T2DM), at *p*-value of 0.05; Table S1: Clinical and biochemical characteristics of patient groups.
