# Supplementary material for: Serum Levels of Nε-(Carboxymethyl)-Lysine in Chronic Kidney Disease and Type 2 Diabetes Mellitus
Source: Biomedicines. 2025 Jul 8;13(7):1672. doi: 10.3390/biomedicines13071672 (PMC12292756; doi:10.3390/biomedicines13071672)
Supplement: Supplementary file 1 [file biomedicines-13-01672-s001.zip › biomedicines-3584299-supplementary.pdf]

## Supplementary Matherials

# Serum Levels of $N^{\varepsilon}$-(Carboxymethyl)-Lysine in Chronic Kidney Disease and Type 2 Diabetes *Mellitus*

**Rositsa Tsekovska*, Evan Gatev, Roumyana Mironova, Simona Kerezieva, Siyana Ilieva, Teodora Ilieva, Bilyana Vasileva, Toshimitsu Niwa, Daniela Popova, Vasil Vasilev**

**Table S1. Clinical and biochemical characteristics of patient groups**

| Variable | Study group | | |
|---|---|---|---|
| | $CKD^{+}$ $T2DM^{+}$ | $CKD^{+}$ $DM^{-}$ | $CKD^{-}$ $T2DM^{+}$ |
| N (patients) | 55 | 22 | 21 |
| **Age** (years) | 65 ± 7 | 52.5 ± 6.5 | 52 ± 6 |
| **Sex** (% male) | 50.9% | 54.55% | 52.4% |
| **eGFR (mL/min/1.73 m²)** | | | |
| G1 (≥90) | 7.3% | 13.6% | — |
| G2 (60–89) | 7.3% | 36.4% | — |
| G3a (45–59) | 16.3% | 22.7% | — |
| G3b (30–44) | 14.5% | 9.1% | — |
| G4 (15–29) | 25.4% | 9.1% | — |
| G5 (<15) | 29.1% | 9.1% | — |
| **Proteinuria (g/24h)** | | | |
| normal (<0.15) | 10.9% | 18.2% | — |
| mild (0.15–0.5) | 32.7% | 22.7% | — |
| moderate (0.5–3.5) | 41.2% | 45.5% | — |
| severe (>3.5) | 14.5% | 13.6% | — |
| **Albuminuria (mg/24h)** | | | |
| A1 (<30) | 16.3% | 18.2% | — |
| A2 (30–300) | 36.4% | 31.8% | — |
| A3 (>300) | 47.3% | 50.0% | — |
| **Duration of diabetes (months)** | 156 ± 72 | — | 60 ± 48 |
| **Fasting glucose (mmol/L)** | | | |
| < 6.1 (normal) | 25.45% | 86.36% | 33.33% |
| 6.1 - 6.9 (impaired) | 14.55% | 9.09% | 19.05% |
| ≥ 7.0 (diabetes) | 60% | 4.55% | 47.62% |
| **Postprandial glucose (mmol/L)** | | | |
| < 7.8 (normal) | 56.36% | — | 61.9% |
| 7.8 -11.1 (impaired) | 23.64% | — | 19.05% |
| ≥11.1 (diabetes) | 20% | — | 19.05% |
| **Glycated hemoglobin (HbA1c) (%)** | | | |
| < 5.7 (normal) | — | — | 9.5% |
| 5.7–6.4 (pre-diabetes) | — | — | 23.8% |
| ≥ 6.5% (diabetes) | — | — | 66.7% |

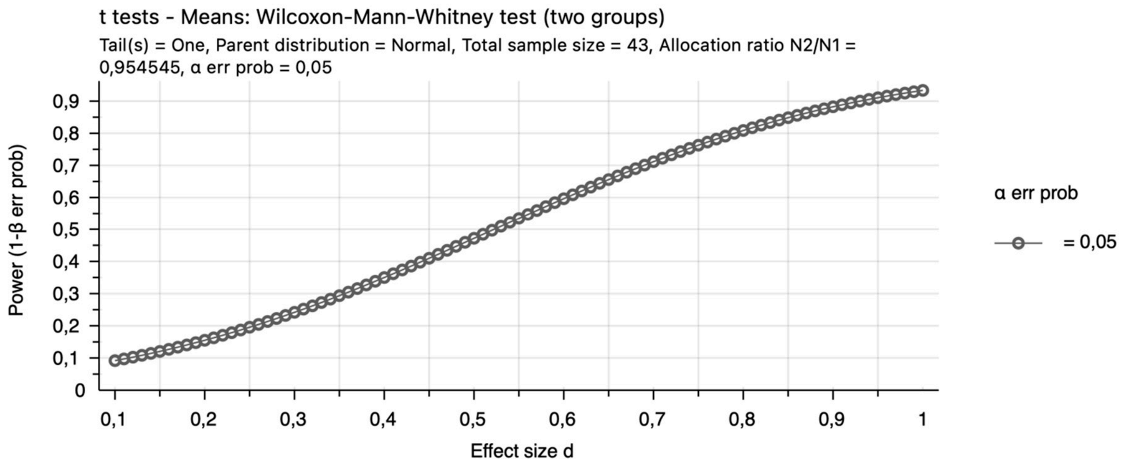


**Figure S1.** Statistical power of Wilcoxon-Mann-Whitney *U*-test for different effect sizes, given samples sizes N1=22 (CKD) and N2 =21 (T2DM), at *p*-value of 0.05.
